# Serum TLR2, TLR4, TLR2/TLR4 Ratio, and ESM1 in Sepsis: A Single-Center Prospective Observational Study of Diagnostic and Prognostic Associations

**DOI:** 10.3390/diagnostics16213570

**Published:** 2026-10-29

**Authors:** Mustafa Usanmaz, Selim Görgün, Metin Ocak, Metin Yadigaroğlu, Meltem Karslıoğlu, Bahar Büşra Özkan, Hümeyra Demirel, Murat Güzel, Özgür Günal

**Affiliations:** 1 Department of Infectious Diseases, Faculty of Medicine, Samsun University, Samsun 55080, Turkey; mustafa.usanmaz@samsun.edu.tr (M.U.); drmeltemkarslioglu@gmail.com (M.K.); bbaharozkan@yahoo.com (B.B.Ö.); ozgurgop@yahoo.com (Ö.G.); 2 Department of Microbiology and Clinical Microbiology, Samsun City Hospital, Samsun 55080, Turkey; 3 Department of Emergency Medicine, Faculty of Medicine, Samsun University, Samsun 55080, Turkey; metin.ocak@samsun.edu.tr (M.O.); metin.yadigaroglu@samsun.edu.tr (M.Y.); murat.guzel@samsun.edu.tr (M.G.); 4 Department of Microbiology and Clinical Microbiology, Faculty of Medicine, Samsun University, Samsun 55080, Turkey; drhumeyrademiral@gmail.com

**Keywords:** sepsis, biomarkers, inflammation, prognosis

## Abstract

**Background/Objectives:** Sepsis remains a major cause of morbidity and mortality, and reliable diagnostic and prognostic biomarkers are needed. This study investigated the diagnostic and prognostic associations of endothelial cell-specific molecule-1 (ESM1), toll-like receptor (TLR)2, TLR4, and the TLR2/TLR4 ratio in sepsis. **Methods:** In this single-center prospective observational study, serum biomarker levels were measured in 60 patients with sepsis and 23 ambulatory controls and analyzed for associations with the presence of sepsis and in-hospital mortality. Demographic characteristics and comorbidities were comparable between groups. **Results:** No significant differences were observed in ESM1, TLR4, or the TLR2/TLR4 ratio between groups (*p* > 0.05). TLR2 levels were lower in patients with sepsis than in controls (median [IQR]: 0.551 [1.508] vs. 1.394 [8.606], respectively; *p* = 0.056), without reaching statistical significance. Receiver operating characteristic analysis yielded an AUC of 0.636 (*p* = 0.080) for TLR2, with 91.7% sensitivity and 47.8% specificity at a cut-off of ≤3.2129. In-hospital mortality occurred in 36.7% of patients. Serum creatinine levels were higher in non-survivors than survivors (median: 2.30 vs. 0.89 mg/dL; *p* = 0.039), whereas no significant associations with mortality were observed for demographic characteristics, clinical findings, other laboratory parameters, or biomarker levels. **Conclusions:** TLR2 levels were numerically lower in sepsis, but the difference was not statistically significant. Its diagnostic utility requires validation against non-infectious systemic inflammation in clinically relevant control populations. No significant diagnostic or prognostic associations were detected for ESM1, TLR4, or the TLR2/TLR4 ratio in this cohort.

## 1. Introduction

Sepsis is one of the leading causes of mortality worldwide and results from a dysregulated host response that can lead to organ dysfunction. In patients with sepsis, the development of septic shock characterized by a profound drop in blood pressure reduces tissue perfusion pressure and may subsequently cause hypoxia [1]. In the United States, sepsis is recognized as the most common cause of in-hospital deaths and is considered a major public health concern due to its substantial economic burden. Consequently, there is a pressing need for reliable biomarkers that can aid in the laboratory diagnosis of sepsis, as well as in its early detection and prognostic evaluation [2].

Sepsis may be triggered by a wide range of infectious pathogens. The current gold standard in its diagnosis is microbiologic testing, which includes blood culture, bacterial identification, and the characterization of pathogens through biochemical or microscopic techniques. In recent years, alternative technologies such as polymerase chain reaction (PCR)-based methods and mass spectrometry have also been increasingly adopted with the aim of reducing diagnostic turnaround time [3].

Given that the mortality rate in patients with sepsis often exceeds 40%, additional strategies have been developed alongside early screening and identification. Preventing organ injury at an early stage and managing the different phases of septic shock, when present, are known to improve patient outcomes [4,5]. Several biomarkers may assist in the diagnosis of sepsis before significant organ dysfunction occurs. These include lactate levels; markers of the hyperinflammatory phase such as proinflammatory cytokines and chemokines; proteins synthesized in response to infection and inflammation, including C-reactive protein (CRP) and procalcitonin; indicators of neutrophil and monocyte activation; anti-inflammatory cytokines; and cell surface receptors expressed on monocytes and lymphocytes [6,7].

Endothelial cell-specific molecule-1 (ESM1) is a proteoglycan-structured molecule regarded as a potential marker of endothelial cell injury in humans [8]. Inflammatory mediators such as interleukin (IL)-1β and tumor necrosis factor (TNF)-α can enhance ESM1 expression in endothelial cells. This signaling interaction may, in turn, induce the expression of adhesion molecules such as intercellular adhesion molecule 1 (ICAM-1) and vascular cell adhesion molecule-1 (VCAM-1), thereby facilitating leukocyte migration and promoting the inflammatory response [9]. In terms of diagnostic value, ESM1 has been reported to be comparable to procalcitonin for identifying sepsis and assessing its severity. However, as a prognostic biomarker, ESM1 is considered to be superior to procalcitonin in patients with sepsis. Furthermore, ESM1 has been shown to be useful in predicting outcomes in conditions such as hospital-acquired pneumonia (HAP), acute respiratory distress syndrome (ARDS), and renal dysfunction [10,11,12].

After an infectious agent enters the body, innate immune cells recognize pathogen-associated molecular patterns (PAMPs) through pathogen recognition receptors (PRRs) [13]. Toll-like receptors (TLRs), first described by Janeway in the late 1980s, are structures that recognize bacterial cell wall (BCW) components and regulate innate immune and inflammatory responses. TLRs are membrane-bound receptors that also induce the transcription and expression of cytokines, thereby enhancing cellular immune function [14]. In recent years, increasing attention has been directed toward TLR expression levels, their signaling pathways, and their potential role in predicting patient outcomes [13,14,15,16].

The present study aimed to investigate the diagnostic and prognostic significance of ESM1, TLR2, TLR4, and the TLR2/TLR4 ratio in patients with sepsis.

## 2. Materials and Methods

### 2.1. Study Design

This study was conducted as a single-center prospective observational study at Samsun University, Samsun Training and Research Hospital, following approval from the local ethics committee on 14 August 2024 (Approval Code: GOKAEK 2024/14/11).

### 2.2. Study Population

**Figure 1:**
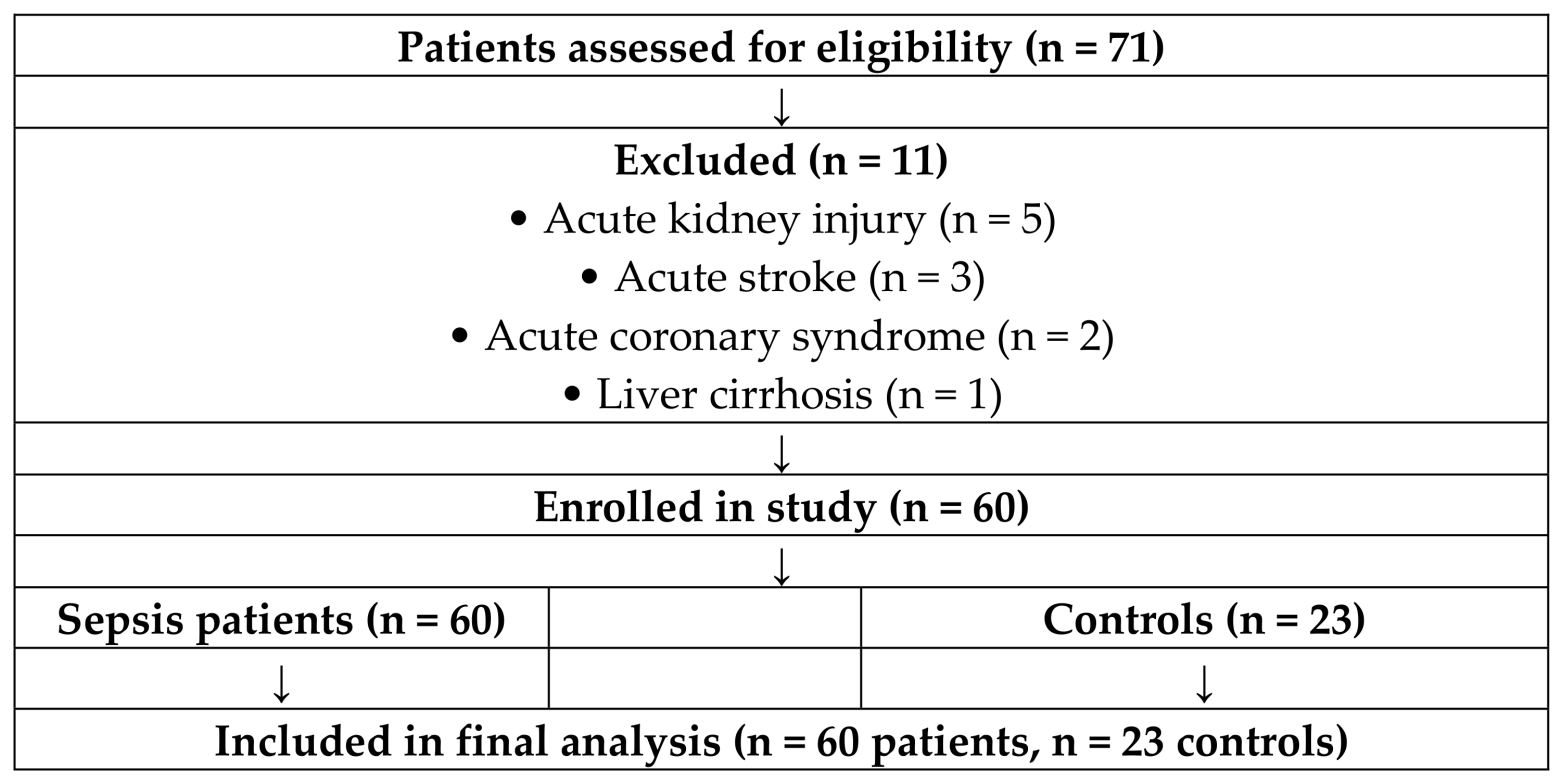
Flow diagram of patient selection.

Patients aged 18 years and older who were diagnosed with sepsis in the ICU were prospectively enrolled in the study between September 2024 and December 2024. Sepsis was diagnosed according to the Sepsis-3 criteria, defined as suspected or confirmed infection accompanied by acute organ dysfunction, represented by an increase in the Sequential Organ Failure Assessment (SOFA) score of at least 2 points from baseline. The study biomarker results were not available to the treating physicians at the time of diagnosis and therefore did not influence the clinical diagnosis of sepsis. Although the Sepsis-3 criteria were used for the clinical diagnosis of sepsis, individual SOFA and APACHE II scores were not systematically recorded in the study dataset. Similarly, the number of patients meeting the Sepsis-3 criteria for septic shock was not documented. Therefore, these data could not be reported. Informed consent was obtained from all participants or their legal representatives. The control group consisted of 23 ambulatory volunteers recruited from the infectious diseases outpatient clinic who met the study eligibility criteria. Acute infection was excluded based on clinical examination. All 23 control subjects initially assessed were included in the final analysis, with no exclusions, sample losses, or missing data. During the enrollment period, a total of 11 patients were excluded: 5 with acute renal failure, 3 with acute stroke, 2 with acute coronary syndrome, and 1 with liver cirrhosis. The study flowchart is presented in Figure 1. All patients received treatment in accordance with the International Guidelines for Management of Sepsis and Septic Shock 2021 [7].

### 2.3. Exclusion Criteria

During the enrollment period, patients with acute kidney injury (AKI), acute stroke, acute coronary syndrome, or liver cirrhosis were excluded from the study. AKI was defined according to the Kidney Disease: Improving Global Outcomes (KDIGO) criteria. Additionally, patients with liver cirrhosis or chronic inflammatory diseases (such as collagen vascular diseases, psoriasis, or inflammatory bowel disease), and those diagnosed as having COVID-19, were also excluded.

### 2.4. Data Collection

Demographic characteristics, vital signs, comorbidities, and laboratory values of the included patients were recorded upon their initial admission to the ICU. Initial antibiotic therapy was started empirically before blood culture results became available. Following the availability of blood culture and antimicrobial susceptibility test results, antibiotic treatment was adjusted accordingly. For arterial blood gas analysis, the second blood sample obtained within the first 24 h was used as the control parameter. Blood samples for TLR2, TLR4, and ESM1 measurements were collected before the initiation of antibiotic therapy. Additionally, all deaths occurring during the index hospitalization were recorded. Comparisons between the patient and control groups were performed with respect to demographic data, vital parameters, laboratory findings, and the levels of ESM1, TLR2, and TLR4. These parameters were also compared between deceased and surviving patients within the sepsis group. The primary mortality endpoint was defined as in-hospital all-cause mortality. Mortality status was assessed throughout the index hospitalization, and all deaths occurring before hospital discharge were recorded. Hospital length of stay was evaluated separately.

### 2.5. Microbiological Analysis

Only blood culture isolates were included in the microbiological evaluation. All 60 patients included in the sepsis cohort had positive blood cultures, confirming the microbiological diagnosis in every case. Cases with polymicrobial bloodstream infections were excluded to avoid potential confounding and duplicate pathogen classification. Coagulase-negative staphylococci (including *Staphylococcus epidermidis*) and *Micrococcus* spp. were considered true pathogens only when recovered from at least two separate blood culture sets and supported by compatible clinical evidence of infection; otherwise, they were classified as contaminants and excluded from the analysis. Pelvic inflammatory disease was diagnosed according to compatible clinical, laboratory, and radiological findings and was considered the source of sepsis when alternative infectious foci were excluded.

### 2.6. Determination of Serum ESM1, TLR2, and TLR4 Levels

Venous blood samples were collected from all participants to determine serum levels of ESM1 (also known as endocan), TLR2, and TLR4. Blood samples were obtained at the time of sepsis diagnosis and before initiation of antimicrobial therapy whenever feasible. Samples were transported to the central laboratory via a pneumatic tube system immediately after collection. Blood samples were centrifuged at 3000 rpm for 10 min within 30 min of collection, and serum aliquots were stored at −80 °C until analysis. Repeated freeze–thaw cycles were avoided. Laboratory personnel performing the ELISA measurements were blinded to the clinical status and outcomes of the participants.

ESM1 levels were measured using an enzyme-linked immunosorbent assay (ELISA) kit (Elabscience, Houston, TX, USA), and TLR2 and TLR4 levels were determined using ELISA kits (FineTest, Wuhan Fine Biotech Co., Ltd., Wuhan, China) according to the manufacturers’ instructions. Optical density was measured using an Infinite 200 PRO microplate reader (Tecan Austria GmbH, Grödig, Austria). The ESM1 assay (Catalogue No. E-EL-H1557) had a detection limit of 9.38 pg/mL and a measurement range of 15.63–1000 pg/mL. According to the manufacturer’s specifications, both the intra-assay and inter-assay coefficients of variation were below 10%. The TLR2 assay (Catalogue No. EH0300) had a detection limit of 0.094 ng/mL and a measurement range of 0.156–10 ng/mL. The TLR4 assay (Catalogue No. EH1033) had a detection limit of 0.188 ng/mL and a measurement range of 0.313–20 ng/mL. TLR4 concentrations below the assay’s detection limit were not considered reliably quantifiable. Therefore, observations with TLR4 values below this limit were excluded from analyses involving the TLR2/TLR4 ratio but retained in all other applicable analyses. According to the manufacturer’s specifications, the intra-assay and inter-assay coefficients of variation were both below 10%. Serum ESM1 concentrations were expressed as pg/mL, whereas TLR2 and TLR4 concentrations were expressed as ng/mL. All samples were measured in duplicate, and the mean value was used for statistical analyses. No additional sample dilution was applied prior to ELISA analysis. The TLR2/TLR4 ratio was calculated by dividing the serum TLR2 concentration by the serum TLR4 concentration for each participant. No transformation was applied before statistical analysis. No missing biomarker data occurred; complete datasets were available for all participants.

### 2.7. Outcome Measures

The study evaluated both the diagnostic and prognostic associations of serum ESM1, TLR2, TLR4, and the TLR2/TLR4 ratio. The primary endpoint was the association of these biomarkers with the presence of sepsis, assessed by comparing patients with sepsis and ambulatory controls. The secondary endpoint was the association of biomarker levels with in-hospital mortality among patients with sepsis. Mortality was defined as death occurring during the index hospitalization. No fixed 30-day follow-up period was used.

### 2.8. Statistical Methods

Statistical analyses were performed using IBM SPSS Statistics version 25.0 (IBM Corp., Armonk, NY, USA) and MedCalc version 20.0 (MedCalc Software Ltd, Ostend, Belgium). The normality of continuous variables was assessed using the Shapiro–Wilk test and descriptive measures of distribution. Continuous variables were summarized as medians with interquartile ranges (25th–75th percentiles) or ranges, as appropriate, and categorical variables were presented as frequencies and percentages. As serum TLR2, TLR4, ESM1, and TLR2/TLR4 ratio values were not normally distributed, between-group comparisons were performed using the Mann–Whitney U test. Categorical variables were compared using the Pearson chi-square test or Fisher’s exact test when the expected cell count was less than 5. Paired comparisons of arterial blood gas parameters between admission and follow-up measurements were performed using the paired-samples *t*-test.

To quantify between-group differences in biomarker levels, rank-biserial correlation coefficients and Hodges–Lehmann location-shift estimates were calculated. Their 95% confidence intervals (CIs) were estimated using stratified nonparametric percentile bootstrapping with 5000 resamples. Potential outliers were evaluated using the 1.5 × interquartile range criterion and were retained in the primary analyses unless attributable to data errors. Biomarker comparisons were considered exploratory, and Holm-adjusted *p*-values were additionally calculated to account for multiple comparisons. Receiver operating characteristic (ROC) curve analysis was performed to assess the diagnostic performance of the biomarkers. Discrimination was expressed as the area under the ROC curve (AUC) with 95% CIs calculated using the DeLong method. Optimal cut-off values were determined using the Youden index, and the corresponding sensitivity and specificity were reported with 95% CIs calculated using the Clopper–Pearson exact method. Exploratory logistic regression analyses were performed to investigate associations between biomarker levels and in-hospital mortality among patients with sepsis. Given the limited number of mortality events, each model included age and one biomarker to reduce the risk of overfitting. Model discrimination was assessed using the AUC, and bootstrap-based internal validation was performed using 500 resamples.

An additional sample-size sensitivity analysis was conducted using the achieved sample sizes to estimate the minimum detectable standardized effect sizes at 80% and 90% statistical power, assuming a two-sided significance level of 0.05 and a parametric independent-group comparison. All statistical tests were two-sided, and *p*-values below 0.05 were considered statistically significant. Given the exploratory nature of the analyses and the limited sample size, the findings were interpreted cautiously.

## 3. Results

A total of 60 patients (72.3%) and 23 controls (27.7%) were included in the study. The median age was 71 (range, 29–93) years in the patient group and 67 (range, 33–88) years in the control group. Among the patients, 36 were male; there were 13 males in the control group. Demographic characteristics and comorbidities were comparable between the patient and control groups (*p* > 0.05 for all).

**Figure 2:**
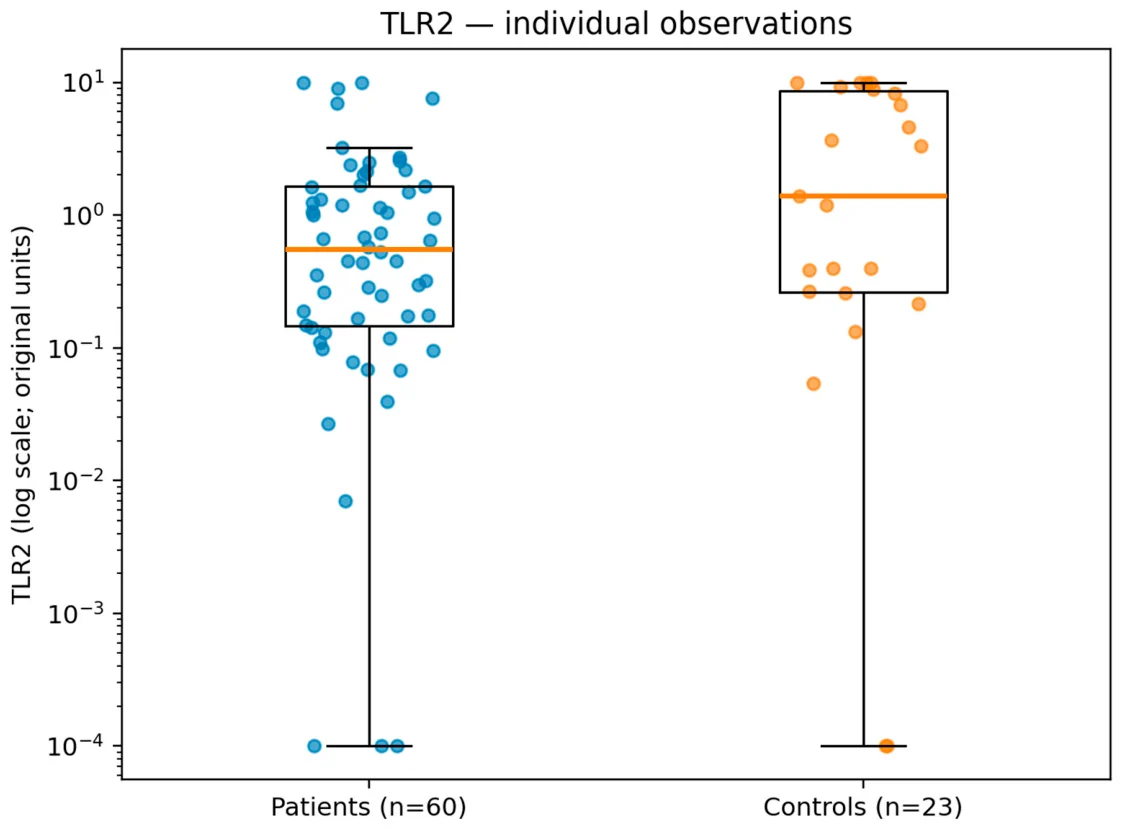
Distribution of TLR2 levels in patients with sepsis and outpatient controls. Individual TLR2 measurements are displayed as points, with boxplots representing the median and interquartile range. The y-axis is presented on a logarithmic scale to improve visualization of the right-skewed distribution and extreme values. Between-group comparisons were performed using the Mann–Whitney U test (*p* = 0.056).

Serum biomarker levels were compared between patients with sepsis and controls. No statistically significant between-group differences were observed for TLR2 (*p* = 0.056), TLR4 (*p* = 0.102), the TLR2/TLR4 ratio (*p* = 0.791), or ESM1 (*p* = 0.618). The corresponding rank-biserial correlation coefficients were −0.272 (95% CI: −0.555 to 0.038), −0.232 (95% CI: −0.496 to 0.044), −0.038 (95% CI: −0.317 to 0.245), and 0.071 (95% CI: −0.209 to 0.348). A sensitivity analysis based on the achieved sample sizes (60 patients with sepsis and 23 controls) indicated that the minimum detectable standardized effect sizes (Cohen’s d) were 0.695 and 0.805 at 80% and 90% statistical power, respectively, assuming a two-sided α of 0.05 and a parametric independent-group comparison. Although TLR2 levels were lower in patients with sepsis than in controls (median [IQR]: 0.551 [1.508] vs. 1.394 [8.606], respectively), the difference showed a borderline trend toward statistical significance (*p* = 0.056; Figure 2). Leukocyte counts were significantly higher in patients with sepsis than in controls (9150 [2100–29,000] vs. 5900 [3700–10,200]; *p* = 0.002). In addition, CRP, total bilirubin, direct bilirubin, and creatinine levels were significantly higher in patients with sepsis than in controls (*p* < 0.001 for all). The sociodemographic characteristics and laboratory parameters of the study population are summarized in Table 1.

**Figure 3:**
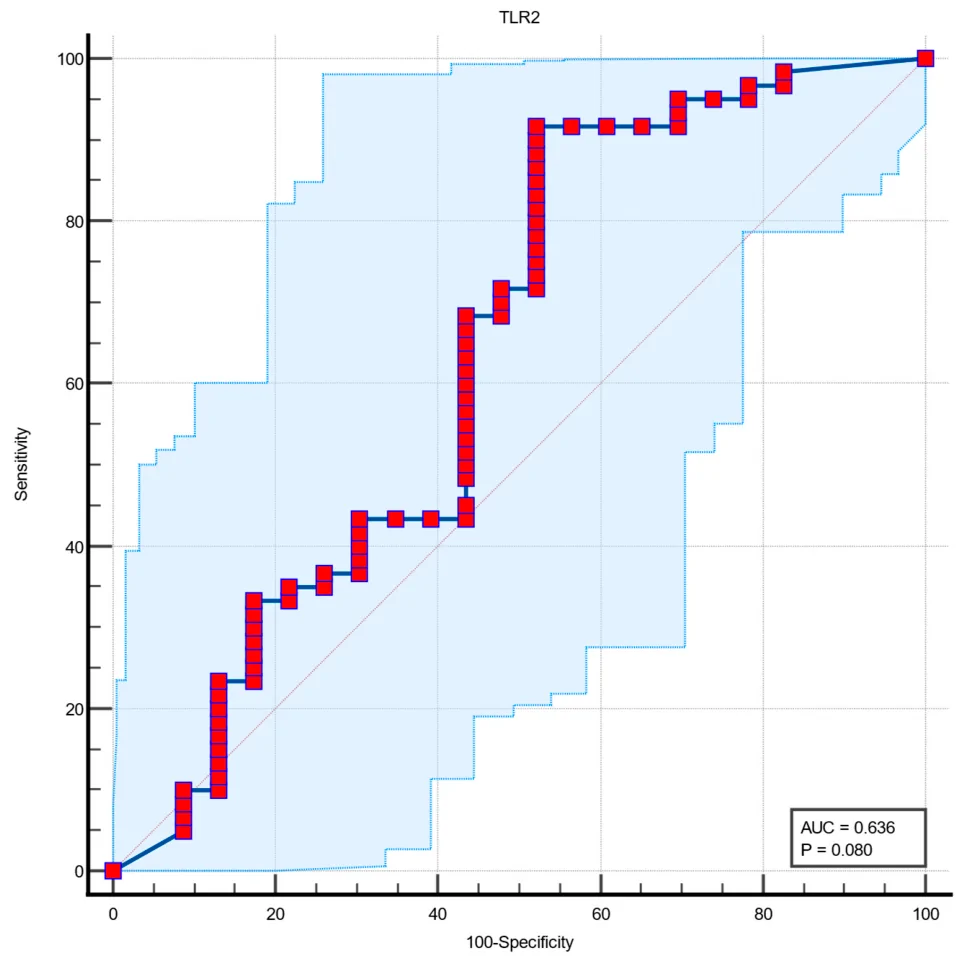
Diagnostic Performance of TLR2: ROC Curve and AUC Analysis.

ROC curve analysis demonstrated that TLR2 had modest discriminatory performance in distinguishing patients with sepsis from healthy controls (AUC = 0.636, 95% CI: 0.484–0.789; *p* = 0.080). The optimal cut-off value determined using the Youden index was ≤3.2129, yielding a sensitivity of 91.67% (95% CI: 81.61–97.24%) and a specificity of 47.83% (95% CI: 26.82–69.41%) (Figure 3).

Among patients with culture-positive sepsis, the most frequently isolated microorganisms were *Klebsiella pneumoniae* and *Acinetobacter baumannii*, each accounting for 23.3% of cases. The most common infection sites were peritonitis (23.3%, n = 14) and urosepsis (21.7%, n = 13). Detailed information on the causative pathogens and infection foci in patients with sepsis is presented in Table 2.

Vasoactive support was administered to 66.7% of patients (n = 40). The recorded agents included dopamine in 16 patients (26.7%), norepinephrine in 14 (23.3%), and epinephrine in 10 (16.7%). Information regarding concomitant or sequential administration, drug doses, and duration of vasoactive support was not systematically available. Antibiotic monotherapy was administered to 24 patients (40.0%). The most frequently used agents were meropenem, piperacillin/tazobactam, and ceftazidime/avibactam, each administered to 5 patients (8.3% of the total cohort). Combination antibiotic therapy was administered to 36 patients (60.0%). Combination antibiotic therapy was applied in 60% (n = 36) of patients, most commonly with piperacillin/tazobactam plus moxifloxacin (11.7%). The mean hospital stay of the patients was 30.55 ± 19.96 days.

Mortality occurred in 36.7% (n = 22) of patients. The median hospital length of stay was 26.5 days (range: 5–102 days). Serum creatinine levels were significantly higher in non-survivors than in survivors (median: 2.30 vs. 0.89 mg/dL, respectively; *p* = 0.039). No significant associations were identified between mortality and demographic characteristics, clinical findings, other laboratory parameters, or biomarker levels.

Exploratory age-adjusted logistic regression analyses did not identify statistically significant associations between the investigated biomarkers and in-hospital mortality. The odds ratios were 0.961 (95% CI: 0.758–1.219; *p* = 0.745) for TLR2, 0.973 (95% CI: 0.877–1.079; *p* = 0.602) for TLR4, and 1.000 (95% CI: 0.994–1.005; *p* = 0.930) for ESM1. Given the limited number of mortality events, these findings should be interpreted cautiously.

A detailed comparison of demographic, vital, and laboratory parameters between the deceased and surviving groups is presented in Table 3.

Arterial blood gas parameters at admission and during follow-up in the patient group are presented in Table 4. Significant improvements in pH, pCO_2_, and lactate levels were observed within the first 24 h of treatment (*p* < 0.05).

## 4. Discussion

Sepsis remains a major global public health challenge, associated with high morbidity and mortality. Recent studies demonstrated that patients with sepsis did not constitute a homogeneous group; rather, they could be classified into distinct sub-phenotypes and endotypes. Factors contributing to these differences include clinical parameters, vital signs, microbial factors and infection sites, as well as biomarkers. These variations have been reported to influence the diagnosis, prognosis, and response to treatment in patients with sepsis [17].

In a 2019 study conducted in Japan involving ICU patients with severe sepsis and septic shock, the most common infection site was the lungs (31.0%), followed by intra-abdominal sources (26.3%), urinary tract infections (18.4%), and soft tissue infections (10.9%). The median patient age was 73 years (IQR: 64–81), and 60.7% were male [18]. In our cohort, the age and sex distribution were comparable to those reported by Abe et al.; however, the two most frequently identified infection sites differed, with peritonitis accounting for 23.3% and urosepsis for 21.7% of cases. Additionally, approximately 25% of patients in our study had sepsis secondary to necrotizing fasciitis or Fournier’s gangrene, representing a notable difference in infection focus. By comparison, Abe et al. reported that soft tissue infections accounted for 10.9% of infection sites [18]. Therefore, our findings present a unique profile of sepsis infection foci, highlighting the particular relevance of skin and soft tissue sources.

In their study, Gatica et al. [19] identified the most common bacterial pathogens in sepsis as *Escherichia coli*, *Klebsiella pneumoniae*, *Staphylococcus aureus*, *Pseudomonas aeruginosa*, and *Streptococcus pyogenes*, with *Candida* species being the most frequently observed fungal pathogens. *Candida auris* has also been recognized as a clinically significant fungal pathogen in ICUs due to its association with high mortality. Mortality related to *Candida auris* infections, which exhibit high antifungal resistance, has been reported to range between 30% and 60%, highlighting the need for urgent evaluation in ICU settings [20]. In our study, the most frequently isolated bacterial pathogens were *Klebsiella pneumoniae* (23.3%), *Acinetobacter baumannii* (23.3%), *Staphylococcus aureus* (13.3%), *Escherichia coli* (11.7%), and *Pseudomonas aeruginosa* (10.0%). *Candida albicans* was isolated in only two (3.3%) patients. Thus, while the bacterial pathogen distribution in our cohort generally aligns with the classic spectrum reported in the literature, the high prevalence of *Acinetobacter baumannii* represents a notable deviation. Furthermore, the absence of fungal species other than *Candida albicans* differentiates our cohort, indicating that *Candida auris* infections, whose importance is increasingly emphasized in the literature, were limited in our study population.

ESM1, a proteoglycan molecule considered a potential marker of endothelial cell injury, has been extensively studied in the literature. In a previous study, serum ESM1 levels of 202 ng/mL demonstrated a sensitivity of 86.7% and specificity of 50% for COVID-19, suggesting that ESM1 could serve as a useful biomarker for both diagnosis and mortality prediction (8). Lassalle et al. [21] reported that patients with sepsis admitted to the ICU with ESM1 levels >3 ng/mL had a high risk of developing lung injury within 72 h, with ESM1 showing 85% sensitivity and 100% specificity for predicting respiratory failure. In our study, no significant difference in ESM1 levels was observed between the patient and control groups. Furthermore, ESM1 levels did not differ significantly between patients who died and those who survived. Our findings contradict previous reports regarding the diagnostic and prognostic value of ESM1, indicating that the clinical utility of this biomarker should be validated in larger and more heterogeneous patient populations. The discrepancy between our findings and previous reports regarding the diagnostic and prognostic value of ESM-1 may be explained by several factors. First, differences in study populations, including patient characteristics, disease severity, comorbidities, and inclusion/exclusion criteria, may have influenced biomarker performance. Second, our relatively limited sample size may have reduced statistical power to detect significant associations. Third, variations in study design, timing of blood sampling, sample handling procedures, and laboratory assay methods could contribute to inconsistent results across studies. In addition, differences in treatment strategies, including the early administration of antibiotics, vasopressors, and supportive care measures, may affect circulating ESM-1 levels and clinical outcomes. Finally, geographic and temporal variations in patient populations, healthcare practices, and pathogen profiles may also account for the observed discrepancies. Therefore, further multicenter studies involving larger and more heterogeneous cohorts are needed to better define the diagnostic and prognostic value of ESM-1 in sepsis.

Recent evidence suggests that circulating TLR2 and TLR4 levels may be altered in patients with sepsis, reflecting changes in innate immune responses during the disease course [22]. In our study, no statistically significant differences were observed between patients with sepsis and controls in TLR4 levels or the TLR2/TLR4 ratio. Although TLR2 levels were lower in patients with sepsis, this difference showed a borderline trend toward statistical significance (*p* = 0.056). This finding may suggest a potential alteration in TLR2-mediated immune responses during sepsis, possibly reflecting changes in the balance between hyperinflammatory and immunosuppressive processes. However, given the lack of conventional statistical significance and the absence of longitudinal measurements, this interpretation remains speculative and requires confirmation in larger prospective studies. TLR2 primarily recognizes peptidoglycan and lipoteichoic acid from Gram-positive bacteria and can also detect components of certain fungi and viruses [23]. TLR4, in contrast, recognizes lipopolysaccharides from Gram-negative bacteria and elicits a strong proinflammatory response during infection [24]. Consequently, the lower TLR2 levels observed in patients with sepsis may reflect alterations in the immune response during sepsis. However, this interpretation remains speculative because the difference did not reach statistical significance and serial biomarker measurements were not performed. Additionally, this difference may be related to the specific patient population or stage of the disease. The lack of prognostic value for the TLR2/TLR4 ratio suggests that these two receptors may be regulated independently during sepsis. In our cohort, ROC curve analysis demonstrated that TLR2 had modest discriminatory performance (AUC = 0.636). At a cut-off value of ≤3.2129, TLR2 showed a sensitivity of 91.67% and a specificity of 47.83%. The relatively low specificity suggests that TLR2 has limited diagnostic utility as a standalone biomarker and should not be used independently to diagnose sepsis.

Our study has several limitations. First, the small sample size and limited number of in-hospital deaths (n = 22) restricted subgroup analyses by disease stage, mortality status, and immunosuppressive phenotype and may have reduced the statistical power to detect prognostic associations. No formal a priori sample size calculation was performed for the primary outcome. Consequently, the limited sample size may have reduced the ability to detect smaller but clinically meaningful differences between groups. Therefore, non-significant findings should not be interpreted as evidence of absent prognostic value. The lack of systematically recorded SOFA and APACHE II scores, septic shock status, and detailed intensive care management data, including renal replacement therapy, duration of mechanical ventilation, and ICU length of stay, limited the comprehensive assessment of disease severity. Furthermore, incomplete information regarding microbiological confirmation, timing of culture collection relative to antibiotic administration, antimicrobial treatment timing and appropriateness, and source control interventions prevented adequate adjustment for potentially important clinical confounders. Since TLR2 and TLR4 levels may be influenced by disease severity, infection characteristics, and treatment-related factors, our biomarker findings should be interpreted cautiously. Second, the single-center design, potential selection bias, and lack of external validation limit the generalizability of our findings. Measurement variability in ELISAs and the absence of serial biomarker measurements may also have affected the assessment of biomarker dynamics. Third, the use of ambulatory controls rather than critically ill patients with non-infectious systemic inflammation prevented evaluation of the ability of TLR2 to distinguish sepsis from other inflammatory conditions, potentially introducing spectrum bias and limiting clinical applicability. Future studies should consider critically ill patients without infection, such as those admitted following major surgery or trauma, as a more clinically relevant comparator group. Additionally, the predominance of Acinetobacter baumannii infections and intra-abdominal and skin/soft tissue infection sources may reflect local epidemiological and referral patterns. Finally, the absence of systematically collected antimicrobial susceptibility data and multidrug-resistant organism classifications precluded a comprehensive assessment of the impact of antimicrobial resistance on clinical outcomes.

## 5. Conclusions

In this prospective cohort of ICU patients with sepsis, serum TLR2 levels showed a nonsignificant tendency toward lower values compared with ambulatory controls (*p* = 0.056). This finding should be considered exploratory and requires further investigation because of the small sample size, control-group selection, clinical heterogeneity, and lack of external validation. In contrast, no statistically significant diagnostic or prognostic associations were detected for ESM1, TLR4, or the TLR2/TLR4 ratio in this cohort. Among the evaluated variables, serum creatinine levels were significantly higher in non-survivors than in survivors, whereas no significant associations with mortality were detected for the investigated biomarkers. No significant associations were identified between mortality and demographic characteristics, clinical findings, or other laboratory parameters. Multicenter studies comparing sepsis with non-infectious systemic inflammation and assessing the incremental diagnostic value of TLR2 beyond established diagnostic tools are warranted.

## Figures and Tables

**Table 1:** Demographic, Clinical, and Laboratory Characteristics of the Patient and Control Groups.

	Patient Group (n = 60)	Control Group (n = 23)	*p*-Value
Age (year) *	71 (29–93)	67 (33–88)	0.294
**Sex**			
**Male**	36 (60)	13 (56.5)	0.773
**Female**	24 (40)	10 (43.5)	
SBP (mmHg) *	95 (80–115)	125 (110–140)	<0.001
DBP (mmHg) *	54.5 (40–75)	90 (70–105)	<0.001
MAP (mmHg) *	67.5 (53–87)	103 (83–117)	<0.001
Heart rate (beats/min) *	94 (82–115)	72 (63–90)	<0.001
Temperature (°C) *	37.4 (36.4–39)	36.4 (36.1–37.5)	0.001
Respiratory rate (breaths/min) *	20 (17–22)	15 (13–17)	<0.001
Saturation (SpO_2_ %) *	89 (83–95)	98 (96–99)	<0.001
GCS *	8 (3–13)	15 (15–15)	<0.001
**Intubation**	42 (70)	0 (0)	<0.001
**Hypertension**	47 (78.3)	17 (73.9)	0.668
**Diabetes mellitus**	20 (33.3)	8 (34.8)	0.901
**Coronary artery disease**	33 (55)	9 (39.1)	0.196
**Chronic kidney disease**	28 (46.7)	15 (65.2)	0.130
**COPD**	18 (30)	7 (30.4)	0.969
**Malignancy**	3 (5)	1 (4.3)	0.901
**Previous stroke**	14 (23.3)	6 (26.1)	0.793
Hemoglobin (g/dL) *	11.7 (8–15.2)	13.2 (10–14.1)	0.049
Leukocyte (/mm^3^) *	9150 (2100–29,000)	5900 (3700–10,200)	0.002
Platelet (×10^3^/µL) *	193.5 (97–394)	199 (134–420)	0.489
Total bilirubin (mg/dL) *	1.65 (0.6–9.2)	0.6 (0.3–1)	<0.001
Direct bilirubin (mg/dL) *	0.95 (0.2–5.3)	0.3 (0.1–0.6)	<0.001
Creatinine(mg/dL) *	1.77 (0.21–4.5)	0.67 (0.34–1.3)	<0.001
ALT (U/L) *	32 (6–144)	29 (18–56)	0.760
AST (U/L) *	34.5 (14–156)	34 (18–55)	0.972
Albumin (g/L) *	33.4 (27–39)	37 (28–42)	0.021
CRP (mg/L) *	117 (3–372)	13 (2–24)	<0.001
ESM1(pg/mL) **	253.01 (101.17)	257.07 (126.13)	0.618
TLR2 (ng/mL) **	0.551 (1.508)	1.394 (8.606)	0.056
TLR4 (ng/mL) **	1.184 (2.210)	2 (3.185)	0.102
TLR2/TLR4 ratio **	0.522 (4.123)	0.579 (2.122)	0.791

SBP: Systolic Blood Pressure, DBP: Diastolic Blood Pressure, MAP: Mean Arterial Pressure, GCS: Glasgow Coma Scale, COPD: Chronic Obstructive Pulmonary Disease, ALT: Alanine Aminotransferase, AST: Aspartate Aminotransferase, CRP: C-Reactive Protein, ESM1: Endothelial Cell-Specific Molecule-1, TLR: Toll-like receptor. * Median (minimum–maximum), ** median (interquartile range [IQR]). Categorical variables are presented as n (%).

**Table 2:** Distribution of Infectious Pathogens and Infection Sources Among Patients with Sepsis.

**Infectious Pathogen**	**Frequency (n)**	**Percent (%)**
*Klebsiella pneumoniae*	14	23.3
*Acinetobacter baumannii*	14	23.3
*Staphylococcus aureus*	8	13.3
*Escherichia coli*	7	11.7
*Pseudomonas aeruginosa*	6	10.0
*Proteus mirabilis*	4	6.7
*Candida albicans*	2	3.3
*Staphylococcus epidermidis*	2	3.3
*Micrococcus*	1	1.7
*Enterococcus faecalis*	1	1.7
Coagulase negative *Staphylococcus*	1	1.7
Total	60	100
**Source of infection**	**Frequency (n)**	**Percent (%)**
Peritonitis	14	23.3
Urosepsis	13	21.7
Necrotizing fasciitis	8	13.3
Fournier’s gangrene	7	11.7
Pelvic inflammatory disease	7	11.7
Psoas abscess	4	6.7
Skin and soft tissue infection	2	3.3
Liver abscess	2	3.3
Ventilator-associated pneumonia	1	1.7
Cholangiosepsis	1	1.7
Bronchitis	1	1.7
Total	60	100

**Table 3:** Comparison of Demographic Characteristics, Vital Parameters, and Laboratory Results Across the Survivor and Non-survivor Groups.

Parameters	Survivors Group (n = 38)	Non-Survivors Group (n = 22)	*p*-Value
Age (year) *	68.5 (30–93)	72.5 (29–88)	0.519
**Sex**			
**Male**	24 (63.2)	12 (54.5)	0.512
**Female**	14 (36.8)	10 (45.5)	
SBP (mmHg) *	95 (80–115)	95 (80–105)	0.152
DBP (mmHg) *	55 (40–75)	50 (40–70)	0.107
MAP (mmHg) *	68 (53–87)	65 (53–82)	0.098
Heart rate (beats/min) *	94.5 (82–115)	94 (86–106)	0.963
Temperature (°C) *	37.5 (36.4–39)	37.4 (36.4–38.5)	0.933
Respiratory rate (breaths/min) *	20 (17–22)	19.5 (17–21)	0.671
**Saturation** (SpO_2_ %) *	89 (84–95)	88.5 (83–93)	0.241
GCS *	8.5 (3–13)	8 (3–13)	0.413
**Hypertension**	30 (78.9)	17 (77.3)	0.879
**Diabetes mellitus**	15 (39.5)	5 (22.7)	0.185
**Coronary artery disease**	24 (63.2)	9 (40.9)	0.095
**Chronic kidney disease**	21 (55.3)	7 (31.8)	0.079
**COPD**	11 (28.9)	7 (31.8)	0.815
**Malignancy**	1 (2.6)	2 (9.1)	0.269
**Previous stroke**	11 (28.9)	3 (13.6)	0.177
Hemoglobin (g/dL) *	11.5 (8–15.2)	12.4 (8.3–14.1)	0.570
Leukocyte (/mm^3^) *	9400 (2100–21,000)	8750 (2200–29,000)	0.247
Platelet (×10^3^/µL) *	180 (97–323)	217.5 (122–394)	0.241
Total bilirubin (mg/dL) *	2 (0.6–9.2)	1.3 (0.2–9.2)	0.073
Direct bilirubin (mg/dL) *	1.1 (0.2–5.3)	0.7 (0.3–4.9)	0.130
Creatinine (mg/dL) *	0.89 (0.21–4.2)	2.3 (0.21–4.5)	0.039
ALT (U/L) *	33 (8–144)	29.5 (6–106)	0.279
AST (U/L) *	35.5 (14–156)	31.5 (19–97)	0.623
Albumin (g/L) *	33.75 (27–39)	33 (29–39)	0.939
CRP (mg/L) *	102 (3–372)	137 (34–330)	0.753
Base excess (mmol/L) **	0.93 ± 11.94	3.34 ± 17.37	0.528
Lactate 1 (mmol/L) **	3.52 ± 2.79	3.37 ± 2.13	0.830
Delta lactate (mmol/L) **	1.71 ± 2.62	1.88 ± 2.07	0.795
Lactate clearance (%) **	31.27 ± 51.29	43.25 ± 35.10	0.336
**ESM1** (pg/mL) ***	252.47 (86.61)	256.42 (190.83)	0.730
TLR2 (ng/mL) ***	0.655 (1.492)	0.422 (1.951)	0.771
TLR4 (ng/mL) ***	1.134 (1.625)	1.195 (2.415)	0.799
TLR2/TLR4 ratio ***	0.507 (2.229)	0.764 (1.025)	0.466

SBP: Systolic Blood Pressure, DBP: Diastolic Blood Pressure, MAP: Mean Arterial Pressure, GCS: Glasgow Coma Scale, COPD: Chronic Obstructive Pulmonary Disease, ALT: Alanine Aminotransferase, AST: Aspartate Aminotransferase, CRP: C-Reactive Protein, ESM1: Endothelial Cell-Specific Molecule-1, TLR: Toll-like receptor. * Median (minimum–maximum), ** mean ± standard deviation (SD), *** median (interquartile range [IQR]). Categorical variables are presented as n (%).

**Table 4:** Comparison of Arterial Blood Gas Parameters Between Initial and Follow-up Measurements in Patient Group (n = 60).

Parameters	Mean ± Standard Deviation	*p*-Value
**Ph-1**	7.34 ± 0.15	0.022
**Ph-2**	7.39 ± 0.069	
**pO_2_-1**	61.13 ± 17.23	0.140
**pO_2_-2**	69.43 ± 40.21	
**pCO_2_-1**	54.86 ± 18.74	<0.001
**pCO_2_-2**	42.49 ± 9.08	
**HCO_3_^−^-1**	29.06 ± 6.15	0.084
**HCO_3_^−^-2**	26.91 ± 6.26	
**Lactate-1**	3.46 ± 2.55	<0.001
**Lactate-2**	1.68 ± 1.31	
**BE-1**	1.82 ± 14.08	0.984
**BE-2**	1.86 ± 9.33	

pO_2_: oxygen partial pressure, pCO_2_: carbon dioxide partial pressure, HCO_3_^−^: bicarbonate, BE: base excess. Data are presented as mean ± standard deviation (SD). Admission and follow-up measurements were obtained from the same 60 patients (n = 60 paired observations). Comparisons were performed using the paired-samples *t*-test.

## Data Availability

Data will be made available on request.

## Abbreviations

The following abbreviations are used in this manuscript:

ESM1	Endothelial Cell-specific Molecule-1
TLR	Toll-like Receptor
PCR	Polymerase Chain Reaction
CRP	C-reactive Protein
IL	Interleukin
TNF	Tumor Necrosis Factor
ICAM	Intercellular Adhesion Molecule
VCAM	Vascular Cell Adhesion Molecule
HAP	Hospital-acquired Pneumonia
ARDS	Acute Respiratory Distress Syndrome
PAMPs	Pathogen-associated Molecular Patterns
PRRs	Pathogen Recognition Receptors
BCW	Bacterial Cell Wall
SBP	Systolic Blood Pressure
DBP	Diastolic Blood Pressure
MAP	Mean Arterial Pressure
GCS	Glasgow Coma Scale
COPD	Chronic Obstructive Pulmonary Disease
ALT	Alanine Aminotransferase
AST	Aspartate Aminotransferase
ROC	Receiver Operating Characteristic
pO_2_	Oxygen Partial Pressure
pCO_2_	Carbon Dioxide Partial Pressure
HCO_3_	Bicarbonate
BE	Base Excess
UTIs	Urinary tract infections
ICU	Intensive Care Unit
ELISA	Enzyme-linked Immunosorbent Assay
SD	Standard Deviation
AUC	Area Under the ROC Curve
